# Multilineage transduction of resident lung cells *in vivo* by AAV2/8 for α_1_-antitrypsin gene therapy

**DOI:** 10.1038/mtm.2016.42

**Published:** 2016-06-29

**Authors:** Julia G Payne, Ayuko Takahashi, Michelle I Higgins, Emily L Porter, Bela Suki, Alejandro Balazs, Andrew A Wilson

**Affiliations:** 1 Center for Regenerative Medicine (CReM) of Boston University and Boston Medical Center, Boston, Massachusetts, USA; 2 The Pulmonary Center, Boston University School of Medicine, Boston, Massachusetts, USA; 3 Department of Biomedical Engineering, Boston University, Boston, Massachusetts, USA; 4 Ragon Institute of MGH, MIT and Harvard, Cambridge, Massachusetts, USA

## Abstract

*In vivo* gene delivery has long represented an appealing potential treatment approach for monogenic diseases such as α_1_-antitrypsin deficiency (AATD) but has proven challenging to achieve in practice. Alternate pseudotyping of recombinant adeno-associated virus (AAV) vectors is producing vectors with increasingly heterogeneous tropic specificity, giving researchers the ability to target numerous end-organs affected by disease. Herein, we describe sustained pulmonary transgene expression for at least 52 weeks after a single intratracheal instillation of AAV2/8 and characterize the multiple cell types transduced within the lung utilizing this approach. We demonstrate that lung-directed AAV2/8 is able to achieve therapeutic α-1 antitrypsin (AAT) protein levels within the lung epithelial lining fluid and that AAT gene delivery ameliorates the severity of experimental emphysema in mice. We find that AAV2/8 efficiently transduces hepatocytes *in vivo* after intratracheal administration, a finding that may have significance for AAV-based human gene therapy studies. These results support direct transgene delivery to the lung as a potential alternative approach to achieve the goal of developing a gene therapy for AATD.

## Introduction

α_1_-antitrypsin deficiency (AATD) is the most common genetic cause of emphysema, affecting an estimated 80–100,000 Americans^1,2^ and resulting in significant morbidity and mortality. Homozygous inheritance of a single base pair mutation (Lys342Glu) is responsible for the most common severe form of the disease, resulting in production of a misfolded protein in hepatocytes, the cell type most responsible for α1-antitrypsin (AAT) production. Misfolding and polymerization of mutant AAT (zAAT) predisposes AATD patients to hepatic cirrhosis and impairs secretion of AAT into the circulation, resulting in protease-antiprotease imbalance in the lung, degradation of elastin by excess neutrophil elastase activity over time, and early-onset panacinar emphysema. Periodic intravenous infusion of human AAT protein to restore circulating AAT levels to a protective threshold, known as augmentation therapy, is the current standard of care for severely affected individuals with lung damage. Although potentially beneficial for patients with AATD-associated lung disease, lifelong augmentation therapy is inconvenient, invasive, and costly,^3^ providing a rationale for alternative treatment approaches.

Delivery of the normal AAT gene via gene therapy is one such potential alternative approach to combat AATD-related progressive lung damage. Many cell types are capable of AAT production, providing researchers with a number of possible target tissues for gene delivery.^4–6^ Early-stage clinical trials utilizing adeno-associated virus (AAV) to target skeletal muscle have demonstrated safety and long-term transgene expression but thus far have been unable to produce the 11 μmol/l (~500–800 μg/ml) of circulating AAT protein considered necessary to protect the lung from neutrophil elastase-mediated lung damage.^7–13^ Induction of transgene expression levels sufficient to replace the second-most abundant circulating blood protein has in practice turned out to be a major technical challenge. Because AAT transport into the lung is an inefficient process,^13–15^ levels of normal AAT reaching the lung are lower than those in the circulation following transgene delivery to distant sites. Indeed, the putative “protective threshold” in the lung epithelial lining fluid is significantly lower than the corresponding threshold in the bloodstream, reflecting these distinct biological compartments.^13^ Together, these factors suggest the possibility that direct delivery of the normal AAT gene to the lung might circumvent one major impediment to successful gene therapy and in so doing provide a therapeutic benefit to patients with AATD.

We have previously reported intratracheal (IT) instillation of lentiviruses to deliver the normal, human AAT (hAAT) gene to resident lung alveolar macrophages (AMs) as a means of protecting against experimental lung injury.^16^ In contrast to integrating retroviral vectors, AAV vectors have a well-established safety profile in multiple human gene therapy trials.^17^ Cross-packaging of recombinant AAV in capsids from recently discovered serotypes allows for optimization of tissue tropism and immunogenicity profiles.^18,19^ Several pseudotyped AAV constructs have accomplished gene delivery to lung cell types, including AAV 5, 6, 8, 9, and 10.^20,21^ AAV2/8 has also demonstrated safety and efficacy in human systemic gene therapy trials for Hemophilia B,^22^ suggesting it as a logical candidate for lung parenchyma-targeted transgene delivery with potential clinical relevance.

Here, we present a strategy combining an advanced recombinant AAV2/8 vector and a lung-directed gene delivery approach to achieve sustained, high-level production of human AAT in mice. Following IT instillation of AAV2/8, multiple cell types within the murine lung are targeted and express the delivered transgene for at least 1 year. This approach resulted in lung-localized production of human AAT at levels predicted to be protective in patients as well as attenuation of injury in an experimental model of emphysema.

## Results

### Sustained intrathoracic transgene expression results from intratracheal instillation of AAV2/8

**Figure 1:**
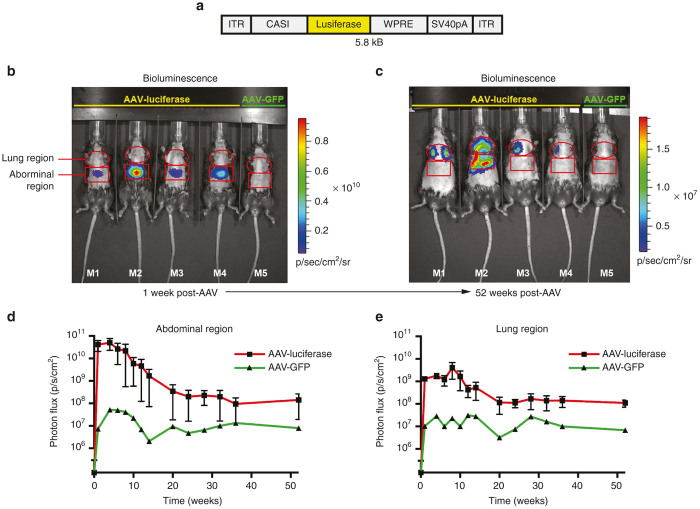
AAV2/8 mediates sustained transgene expression in the mouse abdomen and thorax. (**a**) A schematic representation of AAV8-CASI-luciferase expression vector backbone is shown. The CASI promoter, firefly luciferase transgene, WPRE, and SV40 late-polyadenylation signal are flanked by AAV2 inverted terminal repeats (ITR). (**b**) One week after IT administration, bioluminescence was present in both the abdominal and thoracic regions of AAV-luciferase recipients (*n* = 4, 1 × 10^11^gc; AAV-GFP-treated mouse shown as negative control, *n* = 1), with photon flux (mean ± SD) in the abdominal region exceeding that in the thorax. (**c**) After 52 weeks, abdominal bioluminescence subsided allowing distinct visualization of persistent thoracic bioluminescence. (**d,e**) The bioluminescence kinetic is quantified as total photon flux (photon/s/cm^2^) and isolated according to anatomic region (lung or abdomen) as shown over a period of 52 weeks postadministration of AAV8.

To determine the anatomic distribution and duration of gene expression following IT instillation of AAV2/8, we utilized a vector (hereafter referred to as “AAV8-CASI-luc”; Figure 1a) expressing the firefly luciferase gene under control of the recently published CASI promoter (Supplementary Figure S1).^23^ Intramuscular (IM) injection with this vector results in sustained local expression of firefly luciferase at high levels (Supplementary Figure S2 and ref. 23). One week after a single IT administration of 1 × 10^11^ genome copies (gc) of AAV8-CASI-luc, photon flux was measured in the thoraco-abdominal region of experimental mice (female C57BL/6J, *n* = 4; Figure 1b). Unexpectedly, the initial maximum observed bioluminescence was localized to the abdomen (Figure 1b,d) rather than the thorax, suggesting transduction of cell types outside the lung with this route of delivery. Over time, abdominal bioluminescence declined in three of four IT-treated mice, returning to near-background levels after 24 weeks (Figure 1d). In repeated experiments, this pattern recurred, with 29% of mice exhibiting abdominal bioluminescence which declined to a plateau but did not extinguish, remaining detectable for up to 72 weeks (Figure 1c,d; additional data not shown).

Thoracic bioluminescence was likewise detectable 1 week after IT delivery (Figure 1e) and exhibited a similar kinetic to the abdominal bioluminescence, declining by approximately one log between 10 and 20 weeks after administration before stabilizing and persisting for at least 52 weeks (Figure 1c,e). This durable thoracic transgene expression prompted us to explore further the application of AAV2/8 for lung-directed gene delivery.

### Multiple resident lung cell types contribute to intrathoracic transgene expression

**Figure 2:**
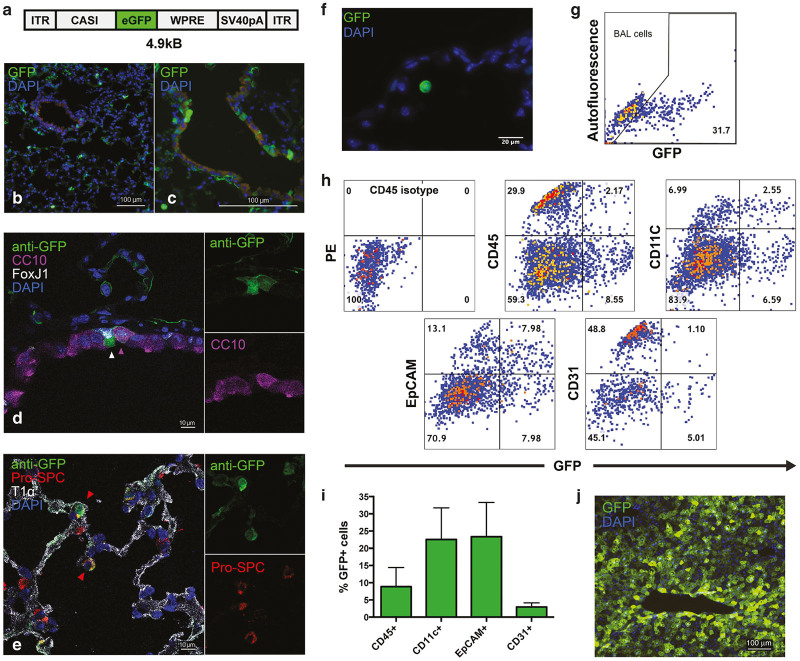
Phenotyping of transduced cells following delivery of AAV 2/8 to the mouse lung. (**a**) A schematic represention of the AAV8-CASI-GFP vector, utilized in these experiments, is depicted. eGFP expression is under the control of the CASI promoter. (**b,c**) Four weeks after IT AAV8-CASI-GFP administration, frozen left lung sections counterstained with DAPI demonstrate multiple GFP+ cells in the airspaces (**b**) and distal airways (**c**). Immunofluorescent staining for CC10 (**d**), Fox J1 (**d**), T1α (**e**), prosurfactant protein C (**e**) and GFP exhibits colocalization of GFP with CC10, FoxJ1, and prosurfactant protein C. Area of inset images in panels **d** and **e** are designated by arrowheads. (**f**) Frozen lung section with DAPI counterstaining demonstrates a GFP+ resident alveolar macrophage within an alveolus. (**g**) Representative flow cytometric analysis of cells from bronchoalveolar lavage performed 4 weeks after IT AAV8-CASI-GFP (gated to isolate live alveolar macrophages); aggregate data (not shown) yielded 32% GFP+ cells (*n* = 4). (**h**) Representative flow cytometric analysis of whole lung digests 4 weeks after IT AAV8-CASI-GFP (*n* = 4) demonstrate GFP+ cells among CD45+, CD11c+, EpCAM+, and CD31+ cell populations. (**i**) Aggregate data from panel H experiment reveals percentages of each cell type found to be transduced (GFP+) by AAV8-CASI-GFP. (**j**) A frozen liver section at low magnification, 4 weeks after IT AAV8-CASI-GFP, illustrates efficient transduction of hepatocytes.

We next moved to identify the phenotype of endogenous lung cells transduced by AAV2/8. Frozen right lung sections harvested 1 month following IT instillation of 1 × 10^11^ gc of AAV8-CASI-eGFP (female C57BL/6J mice, *n* = 3, Figure 2a) revealed the presence of GFP+ cells both within distal bronchioles and alveolar structures (Figure 2b,c). Immunofluorescent staining and confocal microscopy confirmed transduction of multiple endogenous lung epithelial cell types, including club cells (CC10+, Figure 2d), ciliated bronchial epithelial cells (FoxJ1+, Figure 2d) and type II pneumocytes (Pro-SPC+, Figure 2e) as well as nonepithelial cell types (alveolar macrophages, Figure 2f and Supplementary Figure S3). To quantify the transduction of various lung cell types, we analyzed bronchoalveloar lavage (BAL; right lung) and enzymatically digested lung preparations (left lung) from AAV8-CASI-eGFP-treated mice (*n* = 4). Flow cytometric analysis of lung digest samples similarly indicated transduction (GFP positivity) of a variety of cell populations, including epithelial (EpCAM, (23 ± 10%), hematopoietic (CD45, 9 ± 6%), alveolar macrophage (CD11c, 23 ± 9%), and endothelial (CD31, 3 ± 1%) cells (Figure 2h,i). Flow cytometric analysis of BAL cells revealed that a significant percentage of alveolar macrophages were also GFP positive (32 ± 1%; Figure 2g).

AAV8 vectors have been previously demonstrated to be tropic for hepatocytes.^24,25^ To determine whether the intense abdominal bioluminescence observed after IT AAV8-CASI-luc administration represented hepatic transduction, frozen liver sections were likewise analyzed by fluorescence microscopy 1 month following IT AAV8-CASI-GFP. Numerous GFP+ cells exhibiting the characteristic polygonal hepatocyte morphology were observed, consistent with a significant level of hepatic transduction in these experiments (Figure 2j). Antibody staining of liver sections demonstrated that GFP-positive cells in AAV-CASI-eGFP-treated mice stained positive for albumin, confirming hepatocyte transduction in these animals (Supplementary Figure S4).

### IT delivery of AAV-hAAT achieves sustained hAAT secretion in the murine respiratory epithelial lining fluid

To determine whether lung-targeted transgene delivery would result in high level production of a secreted transgene, we next cloned the human AAT gene into the recombinant AAV2 plasmid to create AAV8-CASI-AAT (Supplementary Figure S1). The capacity of AAV8-CASI-AAT to achieve potentially therapeutic local concentrations of hAAT protein was then tested following IT and intramuscular (IM) administration in C57BL/6J mice (*n* = 4 per group). Plasma hAAT levels were measured at baseline and then serially for 28 or 32 weeks, at which time animals were harvested to quantify AAT levels in the BAL.

**Figure 3:**
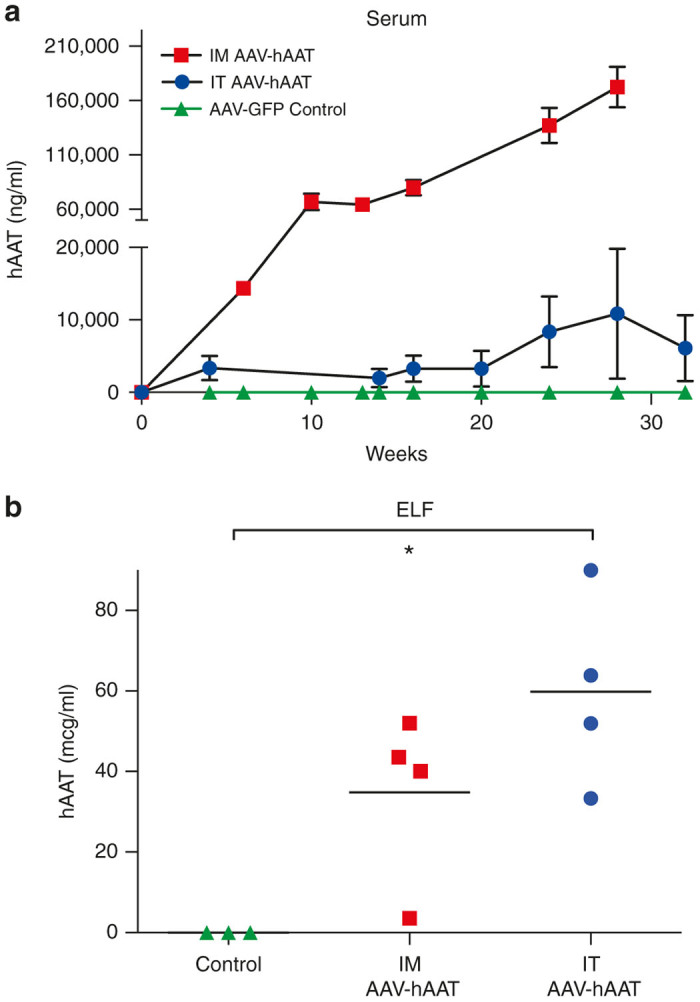
AAV2/8-based expression of human AAT protein in serum and epithelial lining fluid. (**a**) Mice administered 1 × 10^11^gc of AAV8-CASI-hAAT via the IM (*n* = 4) or IT (*n* = 4) route underwent serial measurements of serum hAAT concentration (mean ± SD) by ELISA for 28 or 32 weeks, respectively. (**b**) Mice in each group were harvested at that timepoint for BAL. ELF hAAT concentrations were then quantified by ELISA.

Both IM and IT administration of AAV8-CASI-hAAT resulted in detectible levels of hAAT in the plasma, although neither condition achieved the previously mentioned threshold believed to be sufficient in the circulation to protect against human lung injury (Figure 3a). In contrast to the luciferase expression kinetic outlined in Figure 1, hAAT secretion into the blood following either IM or IT AAT8-CASI-hAAT increased over time (Figure 3a). IM gene delivery led to a 16-fold higher plasma hAAT concentration than IT gene delivery, with a peak of 172 μg/ml (Figure 3a). hAAT was persistently detectable in the plasma following IT administration, potentially reflecting a contribution from multiple cell types, including transduced hepatocytes, to circulating hAAT.

We next harvested the mice to measure levels of secreted hAAT in the epithelial lung lining fluid (ELF). ELF hAAT was measured at 28 or 32 weeks after gene delivery by the IM or IT route, respectively. As anticipated, ELF hAAT levels following IM vector administration were lower than those detected in the circulation at the time of harvest (Figure 3b). In contrast, however, ELF hAAT levels following IT delivery exceeded circulating hAAT levels in these mice, reaching a mean level of 59.8 μg/ml and exceeding the ELF AAT threshold anticipated to protect the lung against injury.^13^

### AAV8-mediated expression of hAAT attenuates elastase-induced emphysema

To confirm the functional relevance of AAV-mediated pulmonary hAAT gene delivery, we employed a murine model of emphysema induced via IT instillation of porcine pancreatic elastase (PPE). This established technique causes elastin destruction and a brisk inflammatory response together with histologic and physiologic features of pan-acinar emphysema including increased airspace size, increased heterogeneity of alveolar size and increased lung compliance that progress over a period of weeks.^16,26,27^

Eight-week-old female C57BL/6J mice were administered 1 × 10^11^ gc of AAV8-CASI-hAAT (*n* = 20) or control vector (AAV8-CASI-GFP, *n* = 15) via the IT route and were maintained for 8 weeks to establish stable transgene expression. A single dose of IT elastase (*n* = 15 per group) or phosphate buffered saline (PBS) control vehicle (*n* = 5, hAAT group only) was then administered 21 days before the time of harvest to induce experimental emphysema and test the protective capacity of hAAT transgene expression (see schematic illustration of treatment, Supplementary Figure S5).

**Figure 4:**
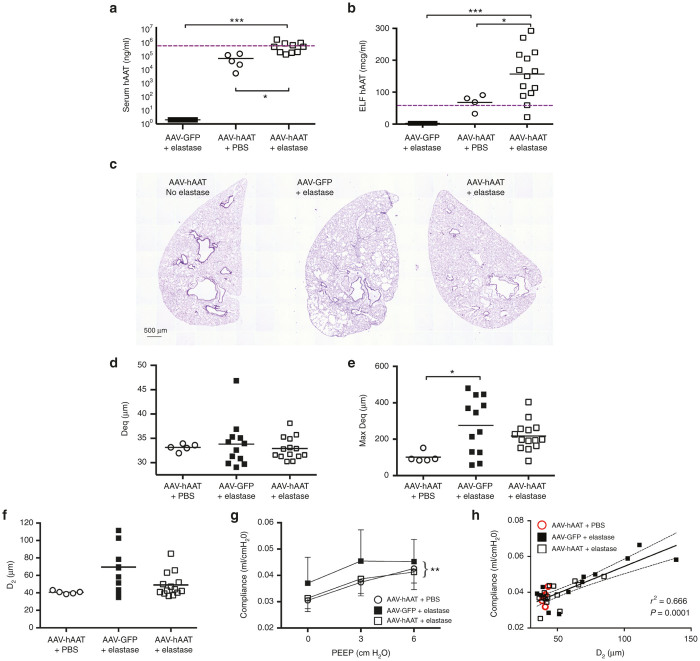
Effects of AAV8-hAAT on lung function and morphometry in the setting of elastase-induced lung injury. (**a,b**) hAAT concentration within the serum and lung epithelial lining fluid 7 weeks after IT AAV8-CASI-hAAT or AAV8-CASI-GFP, and 3 weeks after porcine pancreatic elastase (PPE) or control vehicle. Dashed line represents theoretical therapeutic threshold hAAT concentration (^***^P < 0.001, *P < 0.05). (**c**) Representative axial sections of paraffin-embedded, H&E-stained lung tissue according to vector and PPE treatment group. Additional images are shown in Supplementary Figure S6. (**d–f**) Airspace size is illustrated as equivalent alveolar diameter (Deq), maximum Deq (Max Deq), and area-weighted mean alveolar diameter (D_2_).^26^ (**g**) Lung compliance across a range of PEEP settings was quantified in each treatment group (^**^P < 0.01, see two-way analysis of variance, Supplementary Table S1). (**h**) Demonstration of the relationship between D2 and lung compliance.

As in prior experiments, IT treatment with AAV8-CASI-hAAT resulted in physiologically significant hAAT levels in the plasma, averaging 55 μg/ml 3 weeks after IT elastase instillation (Figure 4a). IT AAV8-CASI-hAAT treatment similarly resulted in high-level hAAT secretion into the ELF, again exceeding the estimated therapeutic threshold for lung protection (Figure 4b). AAV8-CASI-hAAT-treated mice that subsequently underwent PPE-induced lung injury were found to have higher levels of hAAT in both the ELF and serum than PBS-treated controls (Figure 4a,b). This finding contrasts with our previously published lentiviral work^16^ and may reflect the broader tropism of AAV8 or important differences in vector promoter or regulatory element activity.

We next analyzed the degree of elastase-induced lung injury in AAV8-treated mice with histologic and physiologic assays. First, we quantified the effect of PPE on the airspaces (Figure 4c and Supplementary Figure S6) measuring both mean equivalent airspace diameter (Deq, Figure 4d), and maximum equivalent airspace diameter (max Deq, Figure 4e).^28^ We also calculated the D_2_, a validated index which takes into account both alveolar size and heterogeneity (Figure 4f).^28,29^ While mean Deq and D_2_ measurements were marginally larger in the elastase-GFP group compared to noninjured controls, the degree of elastase-induced airspace enlargement at our chosen PPE dose of 0.28U was less than expected, making differences between groups more difficult to detect. The airspace size as measured by maximum Deq, however, was higher in the AAV8-CASI-GFP-elastase group (*P* = 0.023), suggesting a regional elastase-induced airspace enlargement in these animals that was less severe in the hAAT-treated group (Figure 4e, Supplementary Figure S6). Treatment with AAV8-CASI-hAAT resulted in significant protection against elastase-induced alterations in lung physiology as quantified by lung compliance (Figure 4g and Supplementary Table S1). Importantly, histologic and physiologic measures of PPE-induced injury were strongly correlated in this experiment^30^ (Figure 4h) (*P* < 0.0001). Together, these findings suggest that AAV8-mediated hAAT expression diminished the degree of lung injury resulting from PPE instillation in experimental mice.

## Discussion

In this study, we apply a potent AAV8 gene expression system together with a lung-directed administration approach to achieve durable gene delivery to the murine lung. Using fluorescent and bioluminescent reporters, we demonstrate sustained transgene expression for greater than 1 year. Further, immunofluorescent characterization indicates that multiple local cell types in the lung are transduced, including ciliated airway epithelial cells, club cells, type 2 alveolar epithelial cells, vascular endothelial cells, and alveolar macrophages.

Thus far, clinical trials for gene therapy for AATD in humans have demonstrated the safety and feasibility of viral gene transfer for AAT delivery, but have been unable to achieve the high systemic levels of AAT protein required to protect the lung.^4^ Though it is established that significantly lower levels of AAT are necessary within the lung interstitium and ELF than in the peripheral blood to correct protease-antiprotease imbalance and progressive emphysema, AAV-based gene therapy clinical trials have targeted more accessible peripheral tissues. Delivering our AAV8 vector directly to lung parenchyma, we assessed local levels of AAT protein achieved in the lung together with physiologic and histologic changes in a PPE injury model of emphysema.

Our results suggest that IT AAV8 can result in therapeutic secretion of hAAT within the ELF in the absence of a high circulating hAAT concentration, likely due predominantly to local protein secretion by multiple cell types within the lung. While previous studies have provided evidence for AAV8 as an effective pulmonary gene transfer vector,^21,31^ our results provide for the first time detailed phenotyping of transduced lung cell types as well as an illustration of the functional effect of local hAAT gene delivery.

The preservation of lung function in the setting of injury provided by AAV8-directed transgene delivery is an important feature of our studies. The local ELF hAAT secretion levels achieved with our vector surpassed those of previous reports,^21,31^ exceeding the theoretical level required to protect the lung against endogenous elastase activity. For unclear reasons, the protective effects of hAAT transgene expression were more apparent in physiologic measurements of lung mechanics than in histologic quantification of airspace size, potentially representing a limitation in our ability to detect subtle differences in these parameters. Indeed, the tight correlation between D_2_ and lung compliance that we found in these and previous experiments^16^ supports the link between these parameters and diminishes the likelihood that that they might be independently affected by experimental injury.

Interestingly, substantial hepatocyte transduction was observed in the initial weeks following IT administration of AAV8, a finding suggesting early systemic vector distribution. Hepatocytes are known to increase secretion of a variety of proteins as a part of the acute phase response,^32^ potentially contributing to the increases in serum and ELF hAAT secretion demonstrated in our experiments. Liver transduction has been documented after IT administration of AAV vectors,^20^ and our findings are consistent with the avid tropism of AAV8 for hepatocytes.^25,33^ In previous studies of intratracheally delivered AAV vectors, however, hepatic transgene expression was either not reported or vector genome copies in the liver were considered low relative to the lung.^20^ In contrast, AAV8 vector recipients in this experiment displayed early abdominal luciferase expression that exceeded that of the lung region by at least 30-fold. Future consideration of AAV8 for pulmonary gene therapy should take into account the systemic distribution and efficient hepatic targeting of this vector after IT administration. Multiple other tissues not interrogated in our work, including cardiac muscle,^34,35^ skeletal muscle,^19,36^ brain,^37–39^ and retinal^40–42^ tissues are also reported targets of systemic AAV8 and may likewise contribute to systemic transgene expression.

The most common variety of severe AATD in humans, the PI*ZZ form, is marked by not only the deficiency of circulating AAT but also the toxic gain-of-function effects of mutant Z α-1 protein (zAAT), which causes liver disease and has also been speculated to contribute to emphysema pathogenesis.^43–45^ Though few gene therapy approaches targeting AATD address both liver and lung manifestations, promising recent work implements liver-directed AAV9 to achieve miRNA-mediated knockdown of zAAT while simultaneously overexpressing wild-type AAT in PiZ transgenic mice.^6^ IT AAV8, which gains access to both lung and liver tissues after a single treatment, might similarly allow for dual modulation of gene expression while avoiding the high circulating requirement of hAAT for lung protection.

While our results exhibit sustained, therapeutic hAAT expression in C57BL/6J mice with PPE-induced emphysema, this model has limitations. First, due to the embryonic lethality of complete AAT knockout,^46^ no murine model fully recapitulates human AAT deficiency, and murine AAT was expressed concurrently with human AAT in our experiments. Second, while a single dose of IT PPE causes protease-antiprotease imbalance and associated elastin destruction, inflammation and panacinar emphysema in the murine lung, many other factors are likely implicated in the pathogenesis of chronic smoking-related emphysema in humans. Lastly, the degree of emphysema induced by PPE in the control group of our experiment was less severe than in previous experience, potentially decreasing our ability to detect differences between treatment groups.

Careful evaluation of the immune response to IT AAV8 remains an important avenue for future research. While phase 1/2 clinical trials of intravenous AAV8 gene therapy for Hemophilia B have indicated safety in humans, capsid-specific CD8+ T-lymphocyte responses were detected concomitantly with rising aminotransferases necessitating immunosuppressive therapy. Though the clinical significance of anticapsid responses remains unclear, the duration of gene expression may be limited by immune targeting of cells transduced by AAV8.^47,48^ Alternatively, phase 1/2 trials of intramuscular AAV1 for AATD identify a T regulatory mechanism which mediates persistent gene expression despite ongoing anti-capsid responses.^49^ Elucidation of T-lymphocyte responses following IT AAV8 may suggest a mechanism for the observed differences in gene expression duration within the lung and liver, and provide insight into potential risks for human studies.

In summary, our results demonstrate the ability of lung-delivered AAV8 vectors carrying the normal hAAT gene to result in high-level, potentially therapeutic secretion in mice. We provide, to our knowledge, the first detailed phenotyping of lung cell types targeted using AAV8, essential for understanding potential effects of lung-directed gene therapy with this vector in patients. Broadly considered, these results further support the concept of direct lung-targeted transgene delivery for emphysema resulting from AATD.

## Materials and Methods

### Vectors and viral production

Recombinant AAV8 vectors were optimized and produced as previously described in detail.^23^ The AAV8 expression system and viral production, purification and concentration techniques are outlined in Supplementary Methods.

### Animal studies and AAV vector administration

All animal studies were approved by the Institutional Animal Care and Use Committee of Boston University School of Medicine. Female C57BL/6J mice were obtained from Jackson Laboratories at 6 weeks of age . Mice were anesthetized with isoflurane prior to IT, intramuscular or intraperitoneal injections. Thirty minutes prior to vector administration, AAV virus was thawed and diluted in buffer (100 mmol/l sodium citrate, 10 mmol/l Tris pH 8) to a dose of 1 × 10^11^ genome copies in either 100 μl (IT route) or 50 μl (intramuscular route). To administer vector intratracheally, a single dose of AAV was instilled in the posterior oropharynx just above the tracheal entrance using via a blunt-ended, 18-gauge needle. Aspiration of fluid upon inhalation was confirmed in each recipient by investigator visualization. Intramuscular administration of AAV was performed as a single injection into the right vastus lateralis muscle.

### Noninvasive bioluminescence imaging

After isoflurane anesthesia, mice were injected intraperitoneally with 150 μl of D-luciferin (30 mg/ml; Gold Biotechnology, St. Louis, MO). Bioluminescent images were captured 8–16 minutes after D-luciferin injection using the Xenogen IVIS Spectrum, and photon flux was quantified within specified anatomic regions using Living Image software (Caliper Lifesciences, Hopkinton, MA).

### Measurements of lung physiology

Following pentobarbital anesthesia and tracheostomy, mice underwent tracheal cannulation for lung function testing by a computer-driven ventilator (Flexivent; SCIREQ, Montreal, Canada). The forced oscillation technique was used to measure respiratory impedance for estimations of airway resistance and respiratory compliance (C).^50,51^

### Tissue harvesting and analysis

Following euthanasia, the trachea was cannulated and bronchoalveolar lavage was performed by instillation and aspiration of three consecutive aliquots of PBS (500 μl volume per lung). Centrifugation of BAL fluid was performed and supernatant was stored at −80 °C for analysis of hAAT protein and urea content. The BAL cell pellets were immediately resuspended in PBS + 2% fetal bovine serum (FBS) for quantification of GFP-positive cells using flow cytometry. A previously described gating algorithm was used to identify live AMs within BAL cell populations.^23^

To analyze GFP expression within lung tissue, the left lungs of mice were inflation-fixed at 25 cm H_2_O overnight with 4% paraformaldehyde and embedded in Optimal Cutting Temperature compound (OCT). Thin slices of liver tissue were cut using a razor blade before fixation in 4% paraformaldehyde overnight and embedding in OCT. Frozen sections of lung and liver tissue, 5 μm thick, were covered with 4’,6-diamidino-2-phenylindole (DAPI)-containing mounting media (Vector labs, Burlingame, CA) and analyzed by fluorescence microscopy on a Nikon Ni-E Motorized Multichannel upright microscope.

### Enzymatic lung digest

Single-cell suspensions of lung tissue were prepared by enzyme digestion with collagenase A and dispase II (Roche, Basel, Switzerland).^16,52^ Single-cell suspensions of digested cells were stained with PE fluorescence-conjugated anti-mouse monoclonal antibodies against CD45 (BD Biosciences, San Jose, CA), CD11c (eBioscience, San Diego, CA), EpCAM (eBioscience), CD31 (BD Biosciences), and T1α (Biolegend, San Diego, CA). Gating thresholds to identify positively stained cells were determined by staining parallel cell aliquots with antibodies of identical isotype. Propidium iodide (PI; 2 μg/ml; Molecular Probes, Eugene, OR) was added to all samples to exclude dead cells from analysis.

### hAAT ELISA

The concentration of human AAT in plasma and BAL fluid specimens was determined by sandwich enzyme-linked immunosorbent assay (ELISA) (Genway Biotech, San Diego, CA). The lower limit of detection for hAAT was 3.125 ng/ml. After quantitation of hAAT concentration in BAL fluid, hAAT level in lung ELF was calculated using the urea dilution method developed by Rennard and colleagues^53^ (Quantichrom Urea Assay; Bioassay Systems, Hayward, CA).

### Emphysema model

AAV vectors expressing hAAT or GFP were administered via the IT route to adult female C57BL/6J mice (The Jackson Laboratory, Bar Harbor, ME). Eight weeks after treatment, animals received IT instillation of PPE (0.28 U = 30 μg; Sigma-Aldrich, St. Louis, MO) dissolved in 100 μl of PBS or 100 μl of PBS alone (control vehicle). Euthanasia was performed 21 days later at the time of harvest and sample collection.

### Quantification of histology

PFM-fixed lungs were embedded in paraffin by standard methods, sectioned at 5 μm, and stained with hematoxylin and eosin. The equivalent diameters of alveolar airspaces were obtained from digital images and the area-weighted mean alveolar diameter index was computed.^28,29^

### Statistics

For all physiologic and morphometric lung analyses, one-way analysis of variance with *post-hoc* Tukey’s multiple comparison testing or Kruskal-Wallis testing were performed as indicated according to the distribution of results within an experimental group. Two-way analysis of variance with *post-hoc* Tukey’s multiple comparison testing was performed to evaluate differences between compliance, PEEP, and experimental treatment groups (Figure 4d, Supplementary Table S1). Differences in hAAT levels among experimental groups were assessed using one-way analysis of variance with *post-hoc* Tukey’s multiple comparison testing. A *P*-value <0.05 was used to distinguish statistical significance in all studies.

## AUTHOR CONTRIBUTIONS

Conception and design: A.A.W., A.B., and B.S.; Experimental implementation: J.G.P., E.L.P., M.I.H., and A.T.; Analysis and interpretation: J.G.P., A.A.W., A.T., and B.S.; Manuscript drafting for important intellectual contribution: J.G.P. and A.A.W.
