# Supplementary material for: Multilineage transduction of resident lung cells in vivo by AAV2/8 for α1-antitrypsin gene therapy
Source: Mol Ther Methods Clin Dev. 2016 Jun 29;3:16042–. doi: 10.1038/mtm.2016.42 (PMC4926859; doi:10.1038/mtm.2016.42)
Supplement: Supplementary Material [file mtm201642-s1.pdf]

SUPPLEMENTARY MATERIAL

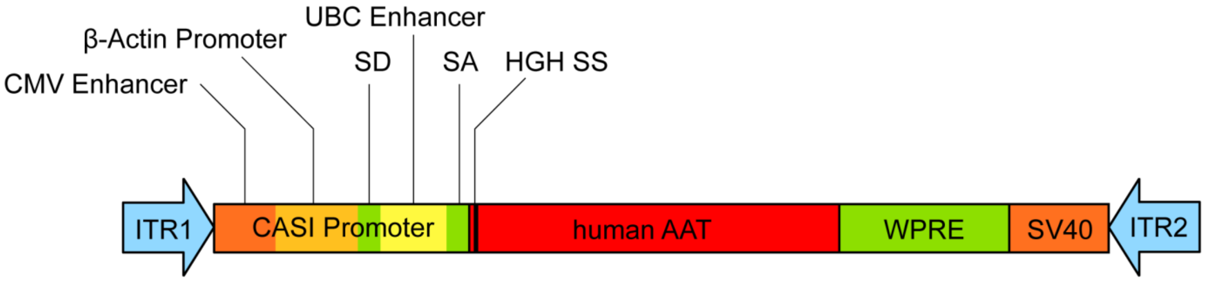

Figure S1: AAV8-CASI-hAAT plasmid map
Schematic representation of the published pVIP transfer plasmid[S1], which features unique restriction sites flanking each modular element (not shown), modified here to deliver the hAAT gene. The 5,446 bp plasmid includes flanking AAV2 ITR sequences together with the synthetic 1.05 kb CASI promoter comprised of the CMV enhancer, chicken β-actin promoter, and UBC enhancer as well as splice donor (SD) and acceptor (SA) sequences. The human alpha1-antitrypsin transgene features a codon-optimized human growth hormone derived signal peptide (HGH SS) to enhance protein secretion. The 3' end of the transgene is terminated with a TAA stop codon, followed by the WPRE element and an SV40 polyadenylation signal.

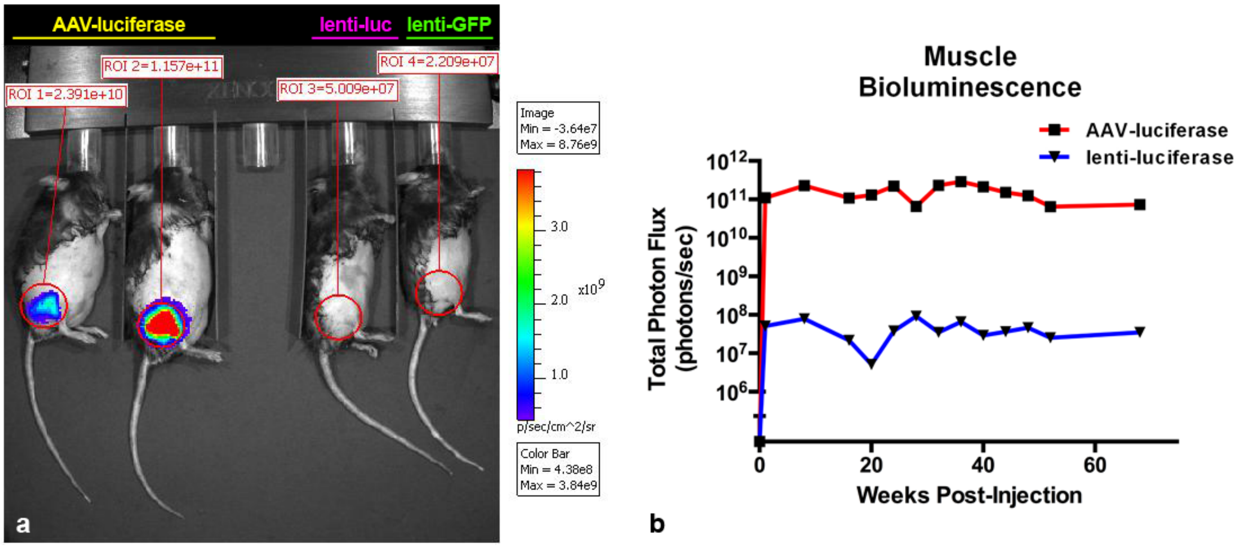

Figure S2: Bioluminescence after intramuscular AAV8-CASI-luciferase
(a) Bioluminescence photon flux imaging of female C57BL6J mice (n=2) 68 weeks after intramuscular injection of 1×10^{11} gc of AAV2/8 encoding CASI-driven firefly luciferase demonstrates significant expression compared to control vector recipients injected with lentiviruses encoding either firefly luciferase or GFP (lenti-EF1αL-luc or lenti-EF1 αL-GFP).[S2]
(b) Quantitation of luciferase gene expression by measured total photon flux demonstrates durable bioluminescence over a 68 week period.

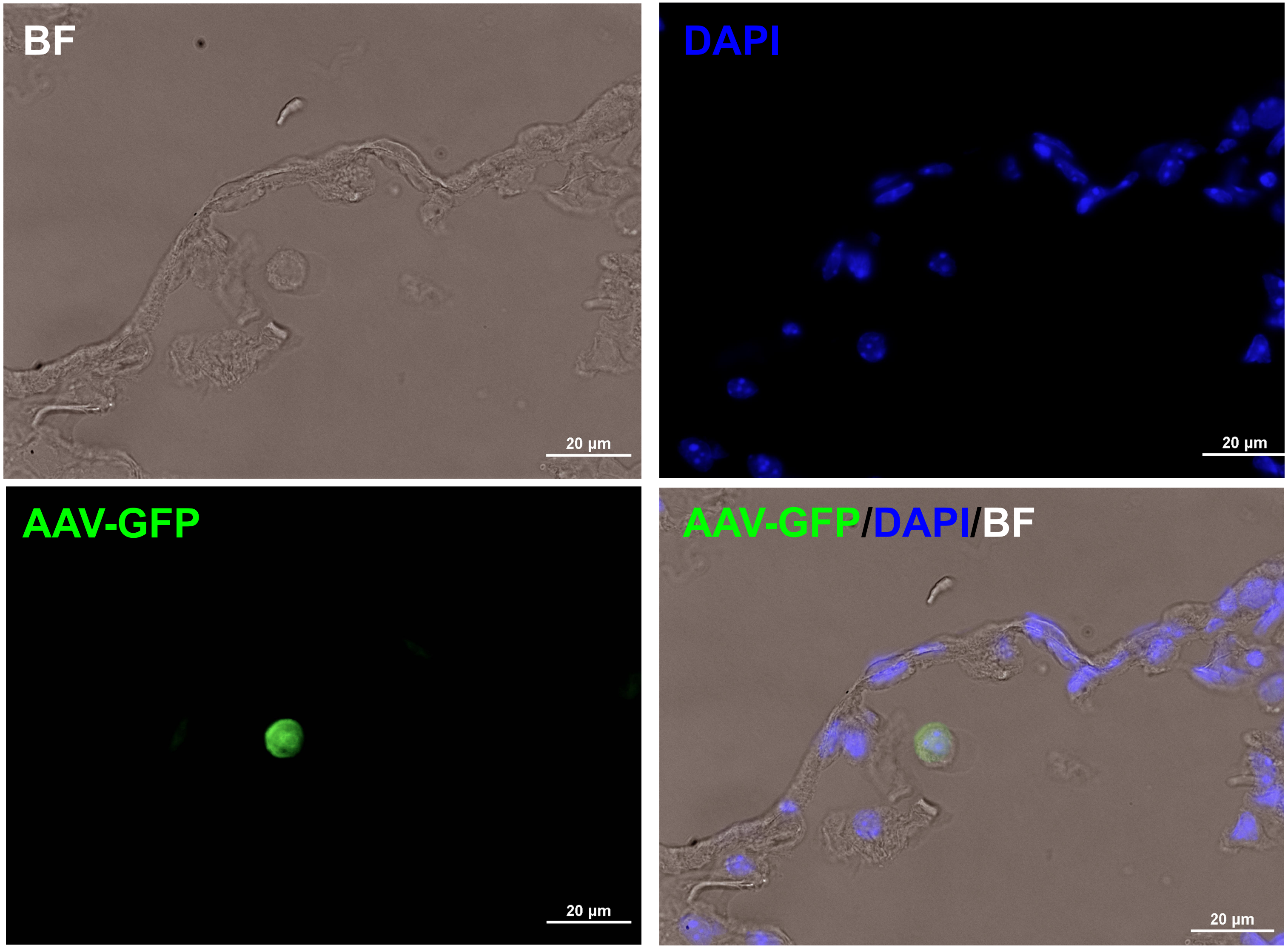

Figure S3: Frozen sections of murine lung 4 weeks after IT instillation of $1x10^{11}$ gc AAV-CASI-GFP demonstrate transduced (GFP positive) cells with the characteristic appearance and location of alveolar macrophages. Images are representative of the experimental group. BF: Bright-field.

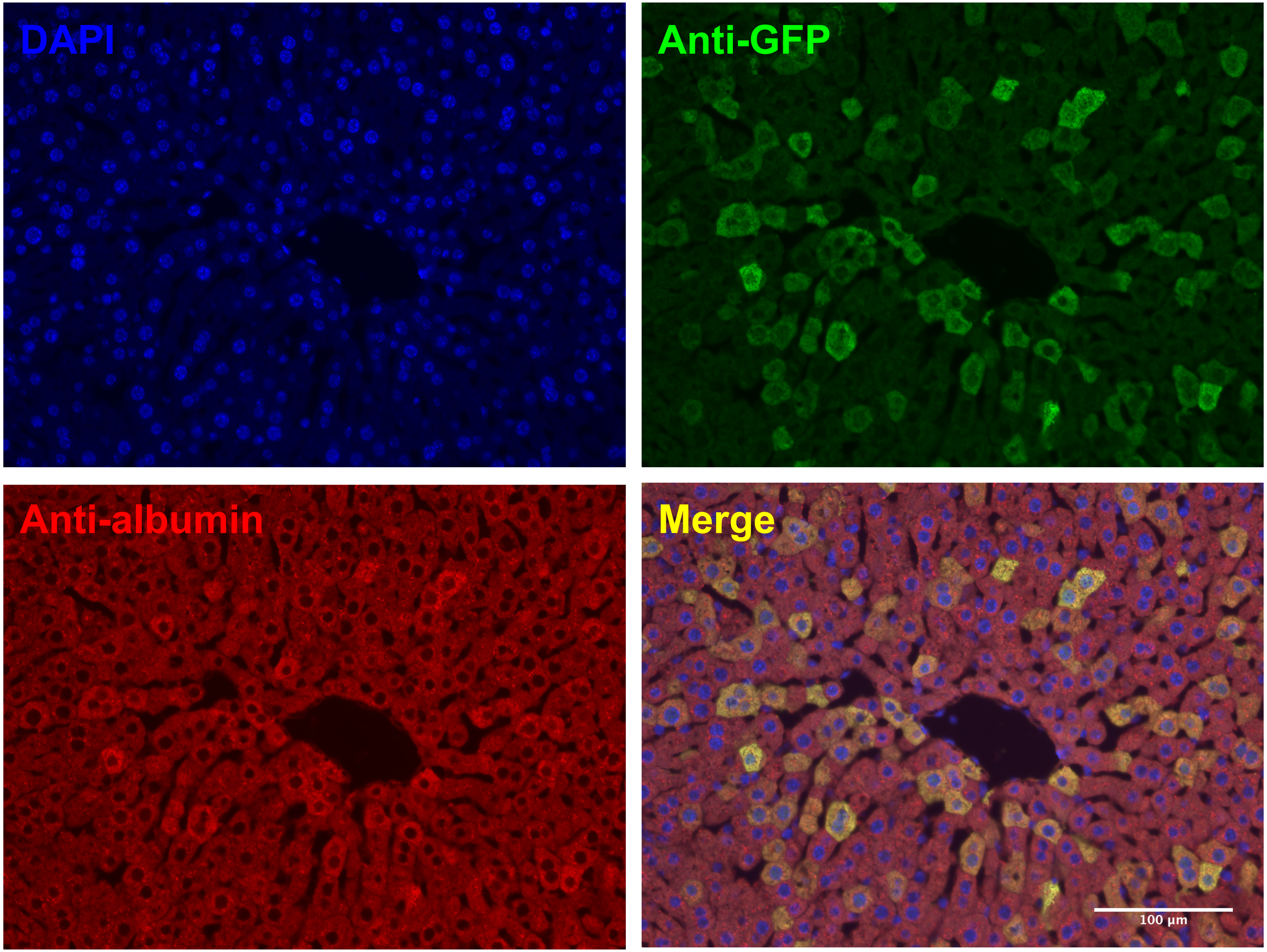

Figure S4: Immunostained frozen sections of murine liver harvested 4 weeks after intratracheal instillation of AAV-CASI-GFP demonstrate colocalization of GFP and albumin.

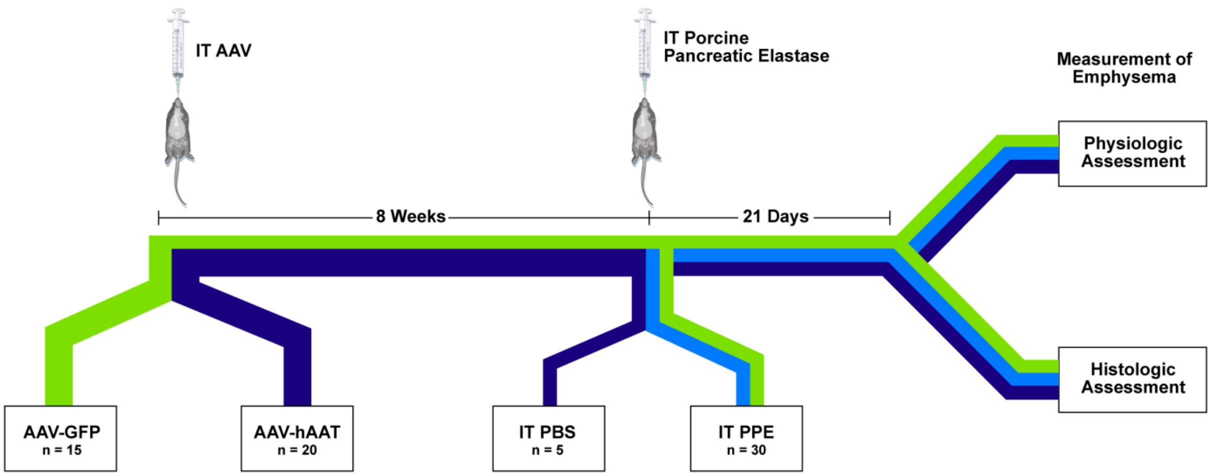

Figure S5: Schematic of Experimental Design for PPE-induced emphysema. Three groups of 8 week old female C57BL/6J mice were administered IT AAV8-CASI-GFP or AAV-CASI-hAAT ($1x10^{11}$gc) before an 8 week incubation period to allow inflammation to subside and establish stable transgene expression. Next, groups were administered either IT porcine pancreatic elastase (PPE; n=15 per group) to induce experimental emphysema or control vehicle (PBS; n=5, vehicle treated mice were taken from hAAT group only as indicated by splitting of the blue bars in the above schedmatic). Twenty-one days later, mice were euthanized for lung function testing and for BAL and histologic analysis of lung tissue.

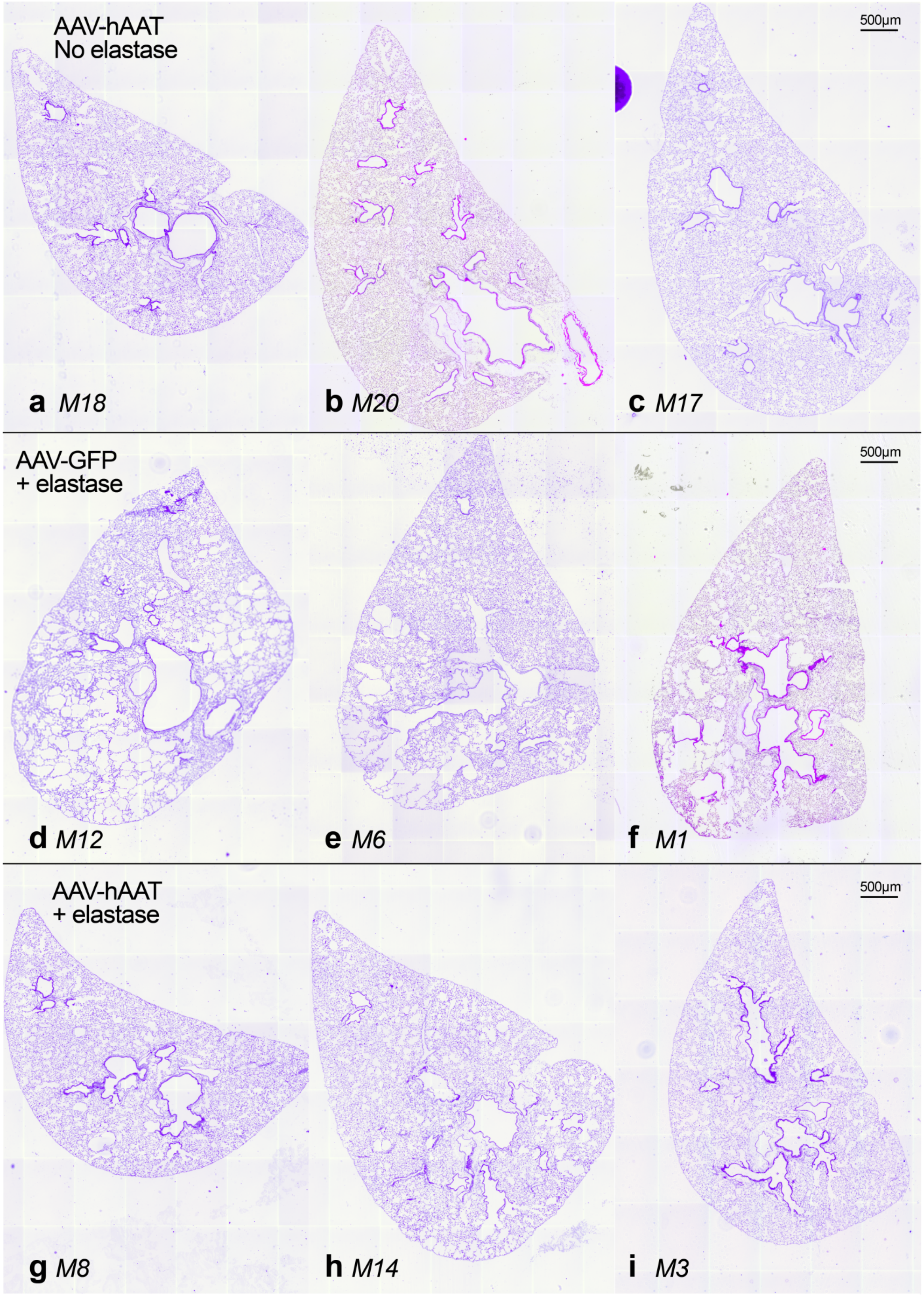
AAV-hAAT
No elastase
500µm
a M18
b M20
c M17
AAV-GFP
+ elastase
500µm
d M12
e M6
f M1
AAV-hAAT
+ elastase
500µm
g M8
h M14
i M3

Figure S6: Representative Lung Sections 21 days after PPE

Representative inflation-fixed axial lung sections, paraffin-embedded and H&E stained, of left lung bases from C57BL/6J mice 21 days after PPE or PBS control vehicle. **(a-c)** Mice treated with AAV-CASI-hAAT and PBS represent negative controls for elastase-induced emphysema and illustrate normal airspace size and heterogeneity. Lung sections from mice treated AAV-CASI-GFP **(d-f)** or AAV-CASI-hAAT **(g-i)** demonstrate regional areas of alveolar destruction 21 days after exposure to IT elastase.

Table S1: Effect of Treatment Group and PEEP on Lung Compliance in Elastase-Induced Emphysema (2-way ANOVA)

Source of Variation	DF	Sum of Squares	% Total Variation	Mean Square	F	P-value
Treatment group	2	0.0006738	9.085	0.0003369	5.56	0.0054
PEEP	2	0.001387	18.71	0.0006936	11.45	0.0001
Interaction	4	6.17E-05	8.32E-01	1.54E-05	0.255	0.9061
Residual	84	0.005089		6.06E-05		

Table S1: 2-way ANOVA was performed to assess the effects of both experimental treatment group and applied PEEP on lung compliance in an elastase-induced emphysema model in C7BL6J mice. Three treatment groups included: AAV8-CASI-hAAT + PBS, AAV8-CASI-hAAT + IT PPE, and AAV8-CASI-GFP + PPE. Applied PEEP was administered at 0, 3 and 6 cmH_20. Both parameters significantly impacted lung compliance, without significant interaction.

Supplementary Methods

Cloning into modular AAV transfer plasmid

The modular AAV transfer plasmid pVIP, developed and described in detail by Balazs and colleagues[S1], was used to construct AAV8-CASI-luciferase, AAV8-CASI-GFP and AAV8-CASI-hAAT vector backbones. Restriction enzymes (NotI, BamHI) allowed for substitution of the luciferase, GFP and hAAT transgenes, while other elements of the vector construct were identical and are illustrated in Figure S1.

AAV virus production and purification

AAV8 was purified from culture supernatants as previously described[s1]. In brief summary, 1.2 x 10^8 293T cells were transfected with 80μg of combined DNA from backbone vector (modified transfer vector in E2) pHELP (Applied Viromics) and pAAV 2/8 SEED (University of Pennsylvania Vector Core) at a 0.25:1:2 ratio using BioT transfection reagent (BiolandScientific). Supernatant collections were performed at 36, 48, 72, 96, and 120 hours post-transfection, when media was filtered through a 0.2μm filter before replacement of 15mL of fresh media.

For PEG precipitation of virus, 75mL of 5X PEG solution (40% polyethylene glycol, 2.5M NaCl) was added to the total volume of supernatant collected and the virus was precipitated on ice for at least two hours. Further purification using cesium chloride fractionation was performed prior to diafiltration, concentration and buffer exchange as previously outlined[s1]. Concentrated virus was suspended in buffer (100 mM sodium citrate and 10 mM Tris pH 8), aliquoted, and stored at -80C.

AAV quantification and functional validation

Purified AAV was quantified by qPCR as previously described[53] with the following modifications. Frozen aliquots of AAV were thawed and diluted ten-fold in digestion buffer containing 10 units of DNAse I (Roche) and incubated at 37°C for 30 minutes. DNAse-digested virus was serially diluted and 5µL of each dilution was used in a 15µL qPCR reaction with PerfeCTa SYBR Green SuperMix, ROX (Quanta Biosciences) and primers designed for the CMV enhancer (5' CMV:AACGCCAATAGGGACTTTCC and 3’ CMV: GGGCGTACTTGGCATATGAT) or the WPRE (5' WPRE: – TGA AAT TTG TGA TGC TAT TGC TTT and 3’ WPRE: CCC CCT GAA CCT GAA ACA TA) Samples were run in duplicate using the Applied Biosystems 7300 Real Time PCR System with the following settings: 1 cycle of 50°C for 2min, 1 cycle of 95°C for 10min, 40 cycles of 95°C for 15s and 60°C for 60s. Virus titer was determined by comparison with a standard curve generated using reference standard consisting of purified AAV2/8 expressing 4E10 antibody. Infectivity of virus aliquots was confirmed *in vitro* by transducing 293T cells with AAV8-CASI-eGFP and performing flow cytometry analysis to quantify %GFP+ cells.

Immunofluorescent staining of Frozen Lung and Liver Sections

After slides were thawed and washed in PBS, antigen retrieval was performed using Antigen Unmasking Solution (Vector Labs) in a microwave oven. For lung sections, blocking was performed with 100mcL donkey serum per section for 30 minutes, prior to application of

primary antibodies. Sections were designated to either receive primary Ab staining for airway cells with mouse anti-human FoxJ1 Ab (ebioscience clone 3C4, 1:300 dilution) and goat anti-human CC10 antibody (T-18 polyclonal Santa Cruz Biotech, 1:1500 dilution) or alveolar staining with hamster anti-T1α Ab (monoclonal ebioscience, 1:1000 dilution) and rabbit anti-prosurfactant protein C antibody (Seven Hills Bioreagents, 1:200 dilution). All sections additionally required chicken anti-GFP antibody staining (Aves labs, 1:500 dilution). For visualization of labeled antibodies, secondary antibodies conjugated to Alexa Fluor 488, 568, and 647 (Invitrogen, 1:300 dilution) and tyramide-conjugated antibodies (Perkin-Elmer; 1:200 dilution) were used. Confocal microscopic imaging was performed using a Zeiss LSM710 metaconfocal laser-scanning microscope.

Frozen liver sections underwent avidin-biotin blocking (Vector Labs) followed by CAS block (Invitrogen) before primary antibody staining with either biotinylated goat anti-Albumin (Abcam, 1:100 dilution) or chicken anti-GFP (Aves Labs, 1:500 dilution) and subsequent secondary antibody staining with Streptavidin-Cy3 (Invitrogen, 1:1000 dilution) or donkey anti-chicken Alexa Fluor488 (Jackson ImmunoResearch, 1:500 dilution). Liver sections were imaged using a Nikon Ni-E Motorized Multichannel upright microscope.

Supplemental References

S1. Balazs AB, Chen J, Hong CM, Rao DS, Yang L, Baltimore D (2011). Antibody-based protection against HIV infection by vectored immunoprophylaxis. *Nature* **481**(7379):81-84

S2. Wilson AA, Murphy GJ, Hamakawa H, Kwok LW, Srinivasan S, Hovav AH *et al*.(2010). Amelioration of emphysema in mice through lentiviral transduction of long-lived pulmonary alveolar macrophages. *J Clin Invest* **120**(1): 379-389.
